# Fabrication of Thixotropic Polymeric Gel System and Its Gelation Mechanism

**DOI:** 10.3390/polym17172397

**Published:** 2025-09-03

**Authors:** Zhilei Zhang, Yuan Geng, Ren Wang, Zhiyuan Yan, Minghao Sun, Sicong Meng, Yan Zhang, Hong Yang, Yaoxuan Li, Yuecheng Zhu

**Affiliations:** 1 CNPC Engineering Technology R&D Co., Ltd., Beijing 102206, China; zhangzhidr@cnpc.com.cn (Z.Z.); wangrdr@cnpc.com.cn (R.W.); yanzhydr@cnpc.com.cn (Z.Y.); sunmhdr@cnpc.com.cn (M.S.); mengsicong@cnpc.com.cn (S.M.); zhangyandr@cnpc.com.cn (Y.Z.); yanghdri@cnpc.com.cn (H.Y.);; 2 School of Petroleum Engineering, China University of Petroleum, Qingdao 266580, China; b22020050@s.upc.edu.cn

**Keywords:** polymer gel, thixotropy, gel strength, high-temperature stability, gelling mechanism

## Abstract

To address the issues of traditional gels in high-temperature reservoir leakage plugging, such as injection–retention imbalance, poor high-temperature stability, and insufficient thixotropy, this study developed a thixotropic polymer gel system via molecular design and component optimization, aiming to achieve excellent thixotropy, high strength, and wide temperature adaptability (80–140 °C) while clarifying its gelation mechanism. First, the optimal polymer was selected by comparing the high-temperature stability and crosslinking activity of AM/AMPS copolymer (J-2), low-molecular-weight acrylamide polymers (J-3, J-4), and AM/AMPS/NVP terpolymer (J-1). Then, the phenolic crosslinking system was optimized: hexamethylenetetramine (HMTA) was chosen for controlled aldehyde release (avoiding poor stability/dehydration) and catechol for high crosslinking efficiency (enhancing strength via dense crosslinking sites). Urea–formaldehyde resin (UF) was introduced to form a “polymer-resin double network,” improving high-temperature compression resistance and long-term stability. Cyclic shear rheological tests showed the gel system had a larger hysteresis area than the polymer solution, indicating excellent thixotropy before gelation. It gelled completely at 80–140 °C (gelation time shortened with temperature). At 120 °C, its viscosity was 7500 mPa·s, storage modulus (G′) 51 Pa, and loss modulus (G″) 6 Pa, demonstrating good shear thixotropy. The final system (1% J-1, 0.3% catechol, 0.6% HMTA, 15% UF) is suitable for high-temperature reservoir leakage plugging.

## 1. Introduction

Polymer gels are typically constituted by a polymeric backbone, crosslinking agents, and a variety of auxiliary materials. Specific functionalities can be engineered through the incorporation of targeted components. Polymer gel systems are primarily classified into three distinct categories [1]. Pre-crosslinked gels are prepared and treated under surface conditions and then injected into the target formation through injection wells to seal the high permeability layer [2,3,4]. Pre-crosslinked gel particles represent a category of preformed gel systems characterized by their millimeter-scale particle size. This plugging technology employs ground-synthesized gel particles as the primary sealing agent. These particles undergo hydration and expansion upon subterranean exposure and are subsequently transported to target leakage zones by differential pressure. The sealing mechanism is achieved through a synergistic combination of bridging, accumulation, and expansive deformation [5]. To enhance the mechanical robustness of the system, inorganic rigid particles may be incorporated during its formulation. These rigid constituents establish a supportive skeletal framework, while the gel components—capable of plastic deformation—occupy the interstitial spaces within this matrix. During the compaction process, this composite structure forms an impermeable barrier, thereby substantially augmenting the overall pressure-bearing capacity of the system [5]. Based on the synergistic mechanism of water-absorbing expansion polymer and rigid skeleton material, Wang et al. developed an FM super gel composite system [6] with a self-expanding physical plugging effect. Through the combination of its own hydration expansion and the support of the rigid skeleton, the material significantly improves the temperature resistance and pressure strength of the leakage plugging system, which can resist high temperatures up to 140 °C and pressure up to 7 MPa.

Underground crosslinked gel materials are used to inject polymer and crosslinking agent into the formation in the form of fluid and then crosslinked to form glue under underground high-temperature conditions to form a plugging layer [6,7,8]. The size matching of the leakage channel of the material is excellent, and the system is in a fluid state before gluing, which can enter the leakage channel of different sizes for plugging under the driving force conditions such as pressure difference. The crosslinking mechanism between polymers and crosslinking agents can be broadly categorized into inorganic metal ion crosslinking and organic crosslinking. Taking the chromium acetate/polyacrylamide system as an illustrative example [9,10], gels formed via ionic bonding exhibit high initial strength; however, due to the relatively low bond energy stability and rapid cross-linking kinetics, such systems are predominantly suitable for applications in shallow, low-temperature reservoirs. To address the limitations of rapid gelation, Chen et al. introduced a delayed crosslinking agent (EA) into the sodium bichromate–sodium sulfite crosslinking system [11]. This modification extended the gelation time to approximately 30 h at 70 °C while maintaining a gel strength of 0.051 MPa. In further efforts to modulate the crosslinking reaction kinetics, Albonico et al. incorporated malonate or hydroxylacetate—functioning as retarders—into a polyacrylamide/chromium-based system [12,13]. Their findings indicated that while such retarders effectively delayed gelation, they concurrently reduced crosslinking network density and consequently led to a deterioration in the mechanical properties of the resulting gel system.

The crosslinking network formed by the covalent bond between organic crosslinkers and polyacrylamide has better adhesive strength and temperature resistance. However, due to the toxicity of formaldehyde (a primary carcinogen classified by the WHO) and phenol, traditional phenolic crosslinking systems have been gradually phased out in industrial applications [14,15]. To meet the environmental requirements of oilfield development, researchers have been actively developing low-toxicity alternatives. Moradi-Araghi evaluated the feasibility of phenol substitutes such as aspirin and salicylate [16], but research on formaldehyde substitutes has remained largely focused on hexamethylenetetramine (HMTA). HMTA releases formaldehyde through controlled thermal decomposition and reacts with amide groups on the polymer to form a gel system characterized by high adhesive strength (with an energy storage modulus exceeding 200 Pa) and high-temperature stability (withstanding temperatures over 120 °C). Sengupta et al. developed a PAM/hydroquinone/HMTA composite system that maintained structural integrity and excellent mechanical properties even after 7 days of aging at 120 °C. Hu et al. developed the gel main agent GN-1 [17], which has good temperature resistance and, based on this research, built a new gel leakage plugging material with high temperature resistance. The gel’s three-dimensional network demonstrated a viscosity retention rate exceeding 85% after being subjected to high-temperature shear conditions (150 °C/100 s^−1^), indicating its exceptional thermal stability.

Non-crosslinked gel plugging technology achieves its sealing function through a molecular self-assembly mechanism, eliminating the need for traditional crosslinkers. Representative systems include cement slurry, the specialized gel ZND, and supramolecular leak-plugging materials. Cement slurry is widely employed as an inorganic plugging agent; due to its rapid solidification characteristics, it is often used to seal severe loss zones such as carbonate rocks or gravel layers. Through molecular design, specialized gel systems introduce functional monomers into the polymer backbone, enabling the formation of a dynamic physical crosslinked network. These systems achieve enhanced performance by synergistically compounding rigid and flexible materials [17]. Based on the synergistic mechanisms of structural fluid theory and supramolecular chemistry, Wang et al. developed the ZND specialized gel plugging system [18]. By introducing functional monomer units into the macromolecular chain, the material builds a dynamic physical crosslinked network by interacting with non-covalent bonds such as hydrogen bonds and van der Waals forces, forming a supramolecular structure with reversible reconfiguration characteristics. Its unique viscoelastic response and shear thinning resistance enable it to maintain high apparent viscosity during seepage. When the system enters the leakage channel, the molecular chains gradually increase the network density through the hysteresis entanglement effect, and finally form a high strength blocking barrier. Combined with ZND gel, strength regulator TD-1 and conventional bridge plugging particles, the drilling plugging system developed by Su et al. can effectively plug surface cracks (pressure exceeding 3.6 MPa) [19], and its mechanism lies in the synergistic stress dissipation effect of dynamic network and rigid particles.

Shear thixotropic polymer gels need both moderate crosslinked network structure and high temperature resistance. In order to meet the engineering requirements of high-temperature oil reservoirs, high-temperature resistant crosslinking agents containing rich carbon–carbon double bond active groups can be used to improve the thermal stability of materials by forming high-density crosslinking networks. This kind of gel system can present a low viscosity fluid state in the flow state, so as to facilitate injection into the formation leakage channel [20]. After the injection reaches the target layer, the shear force disappears, resulting in significant viscosity, which is more likely to stay in the target layer. The gel system can form high-strength gel by a biochemical crosslinking reaction at high temperature, which can effectively seal the lost layer [21]. This material system can not only resist the dilution of formation fluid but also delay the gravity settlement process by adjusting the gel strength, which provides an effective solution for the loss control of complex formation. To meet the engineering requirements of high conductivity fracture plugging and efficient spread of remaining oil, Ge et al. enhanced the strength of a thixotropic gel system with sodium montmorillonite particles [22,23]. The system achieves the balance between injection and retention by optimizing rheological parameters. At different shear rates, the viscosity characteristics match the formation conditions, and the shear dilution behavior effectively reduces the injection pressure.

At present, the gel rheological control systems which are widely used are mainly composed of long-chain polymers and layered silicate minerals. Through the hydration expansion and dispersion of rheological regulator and the bridging effect of the long-chain polymer, the thixotropic three-dimensional spatial grid structure is formed [24]. The results show that this kind of system presents high plastic viscosity in the pre-gluing stage, and the pipe adhesion phenomenon easily occurs during the pumping process, which makes it difficult for fluid to uniformly move into the leakage space and further affects the full filling effect. When the system enters the leakage channel after shear failure, the recovery rate of the structure is low, which limits its effective residence in the target region [8]. These limitations restrict the application of traditional rheological systems in complex conditions. The development aims to address three major limitations of conventional gels in high-temperature reservoir loss control—specifically, injection–retention imbalance, inadequate thermal stability, and insufficient thixotropy. By incorporating functional components with specific rheological response characteristics [25], the thixotropic behavior of the system can be significantly enhanced. Furthermore, optimizing the material formulation through molecular design theory enables efficient energy utilization during the formation and breakdown of network structures, representing a critical direction for future advances in the field. Through these strategies, a novel thixotropic polymer gel system has been developed that exhibits excellent thixotropy, high strength, and broad temperature adaptability (80–140 °C). The gelation mechanism has been elucidated, providing crucial technical support for effective fluid loss control in high-temperature reservoirs.

This study involves a systematic approach to develop an optimized gel system: (1) screening polymer matrices by comparing the high-temperature stability and crosslinking activity of an AM/AMPS copolymer (J-2), low-molecular-weight acrylamide polymers (J-3, J-4), and an AM/AMPS/NVP terpolymer (J-1) to identify the optimal polymer; (2) optimizing the crosslinking system using controlled-release aldehyde-based crosslinkers (HMTA) and efficient phenolic crosslinkers (catechol) to resolve the conflict between stability and crosslinking efficiency; (3) constructing a dual-network structure by incorporating urea–formaldehyde resin (UF) to establish a “polymer-resin dual network”, enhancing high-temperature compression resistance and long-term stability; and (4) validating the performance and mechanism through rheological tests, structural characterization (FTIR, NMR, SEM), and high-temperature gel performance experiments to demonstrate thixotropy, strength, and temperature adaptability and elucidate the gelation mechanism.

## 2. Results and Discussion

### 2.1. Preparation of Polymer Gel System

**Figure 1:**
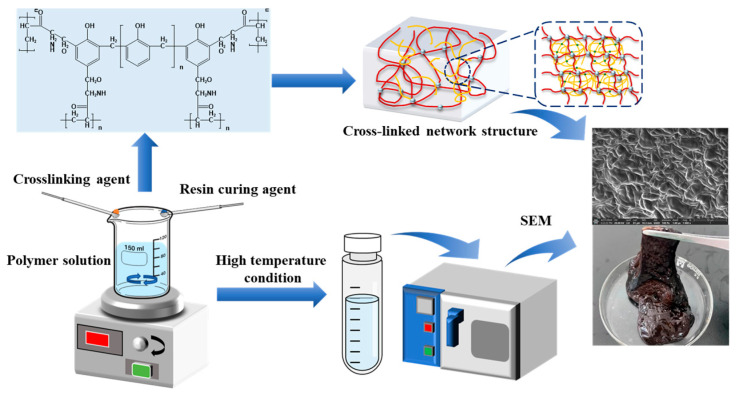
Preparation of polymer gel system.

The thixotropic polymer gel plugging system developed in this study comprises three main components: a polymer, an organic phenol–formaldehyde crosslinking system, and a urea–formaldehyde resin curing agent. The preparation process, illustrated in Figure 1, involves the following specific steps: First, 10 g of polymer powder was gradually added to 1 L of deionized water under continuous magnetic stirring to prevent agglomeration and ensure the formation of a homogeneous dispersion. The solution was then allowed to swell statically at room temperature for 24 h, resulting in a 1.0 wt% polymer stock solution. The polymer concentration can be quantitatively adjusted based on experimental requirements. Subsequently, a specified amount of the organic phenol–formaldehyde crosslinking agent and resin curing agent was introduced into the polymer solution under continuous stirring. After stirring for 30 min, a thixotropic polymer gel precursor solution is obtained, which is then transferred into high-pressure, high-temperature resistant bottles. The solution is placed in a constant-temperature oven at 80–140 °C to complete the gelation process. This standardized procedure ensures full extension of the polymer molecular chains and guarantees reproducibility in the subsequent crosslinking reaction.

### 2.2. Construction of Polymer Gel System

#### 2.2.1. Polymer Type and Concentration Preference

The structural characteristics of the polymer are decisive for the performance of the gel system. Research indicates that the distribution of functional groups along the polymer backbone directly affects crosslinking kinetics. A higher density of specific functional groups [9], such as carboxyl (–COOH) and hydroxyl (–OH), can substantially enhance the reactivity of the crosslinking process and the density of the resulting network. From the perspective of molecular design, the molecular weight of the polymer is determined by the monomer conversion rate during polymerization. Increased monomer conversion leads to higher molecular weight and more pronounced chain extension. The degree of hydrolysis is positively correlated with the carboxyl group content in the molecular chain, which is closely associated with the crosslinking mechanism. The gel system developed in this work employs an organic phenol–formaldehyde crosslinking agent with amide (–CONH_2_) groups as active sites. However, its hydrolysis degree ranges from 10% to 30%, limiting its ability to regulate the gel network formation. When the hydrolysis degree of the polymer falls below a critical value, its hydration capacity is significantly reduced, consequently slowing the crosslinking reaction. This is manifested as prolonged gelation time of the system.

The J-1 polymer was synthesized via terpolymerization of acrylamide (AM), 2-acrylamido-2-methylpropane sulfonic acid (AMPS), and N-vinylpyrrolidone (NVP). By controlling the monomer molar ratio, initiator concentration, and reaction temperature, the molecular weight and degree of hydrolysis (DH) were precisely regulated. The weight-average molecular weight (M₍w₎) was maintained between 3.0 × 10^6^ and 5.0 × 10^6^ Da, and the DH was controlled within 15–20%. This specific DH range ensures appropriate hydrophilicity and charge density, facilitating optimal water solubility and rheological behavior—essential for subsequent functional performance. In comparison, the J-2 polymer was prepared via copolymerization of AM and AMPS only. Its molecular weight was adjusted to a range similar to J-1 (3.0 × 10^6^–5.0 × 10^6^ Da), while the DH was set at 10–15%. This design allows systematic evaluation of the influence of DH on the properties of AM/AMPS-based polymers under controlled conditions, highlighting the role of functional groups such as carboxyl and sulfonate in structure–property relationships. Polymers J-3 and J-4 are low-molecular-weight linear systems based on AM, with a DH of 20–25% achieved through controlled hydrolysis. Their molecular weight is significantly lower than that of J-1 and J-2, as confirmed by gel permeation chromatography (GPC). This low molecular weight enhances molecular diffusion and interfacial adsorption kinetics, making these polymers suitable for applications requiring rapid migration.

To comprehensively analyze the chemical composition, functional groups, and molecular chain structure of the J-series polymers, Fourier transform infrared spectroscopy (FTIR) and proton nuclear magnetic resonance spectroscopy (^1^H NMR) were employed. FTIR provides rapid qualitative identification of characteristic functional groups based on the position, intensity, and shape of key absorption peaks. In contrast, ^1^H NMR enables quantitative determination of comonomer composition and the degree of hydrolysis (DH) by analyzing the chemical shifts and integration areas of proton signals. Together, these techniques form a complementary approach for detailed structural characterization.

**Figure 2:**
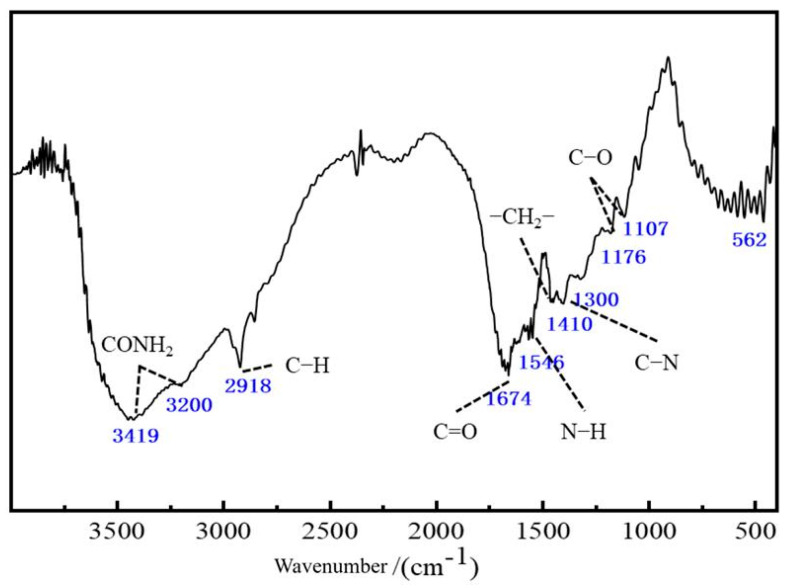
Infrared spectrum of polymer J-1.

The FTIR spectra of J-1 to J-4—obtained from vacuum-dried powders following the method in Section 3.2.5—are presented in Figure 2. Taking J-1 as a representative example, multiple characteristic absorptions are evident. A broad and strong peak at 3419 cm^−1^ corresponds to N–H stretching from amide groups, with its broadening indicating hydrogen bonding and high acrylamide content. Nearby, the absorption around 3200 cm^−1^ is attributed to overlapping O–H (from –COO^−^) and S–O (from –SO_3_^−^) stretching vibrations, confirming the presence of hydrolyzed carboxylate and AMPS-derived sulfonate groups. Aliphatic C–H stretch appears at 2918 cm^−1^, while amide-related bands are observed at 1674 cm^−1^ (Amide I) and 1546 cm^−1^ (Amide II). Additional features include the –CH_2_–CH_3_ deformation at 1410 cm^−1^, C–N stretching of NVP at 1300 cm^−1^, carbonyl vibration at 1176 cm^−1^, and sulfonate-specific S=O symmetric stretch at 1107 cm^−1^ with S–O bending at 562 cm^−1^.

**Figure 3:**
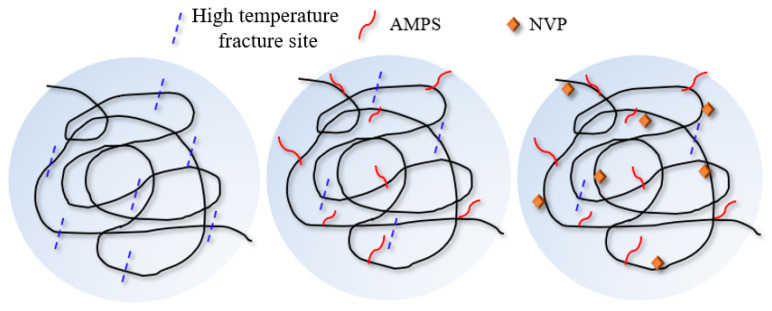
Schematic diagram of the microstructure of polymer J-1.

Based on the above FTIR analysis, it is evident that polymer J-1 exhibits structural characteristics of both AMPS and NVP, as shown in Figure 3. Combined with Figure 3 and the TGA thermal analysis results (degradation temperature 347 °C), it can be seen that the exceptional thermal stability of this terpolymer (AM/AMPS/NVP) is attributed to its unique five-membered ring rigid structure. The enhanced high temperature resistance of the polymer is primarily attributed to two key mechanisms: First, the AMPS side groups hinder the thermal hydrolysis of amide groups through steric hindrance, effectively preventing the formation of carboxyl groups. Second, the five-membered ring configuration dissipates molecular energy, reducing the probability of molecular chain scission caused by thermal vibrations. This significantly minimizes high-temperature breakage sites along the polymer backbone.

**Figure 4:**
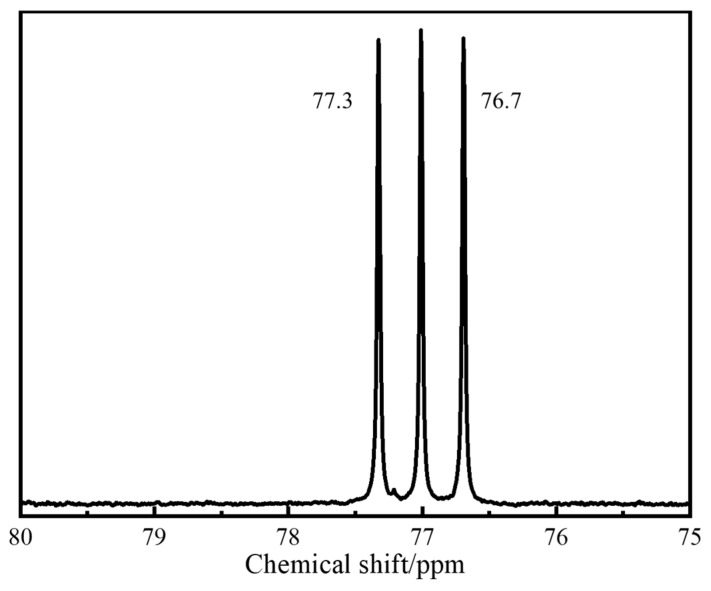
^13^C NMR spectra of polymer J-1.

**Figure 5:**
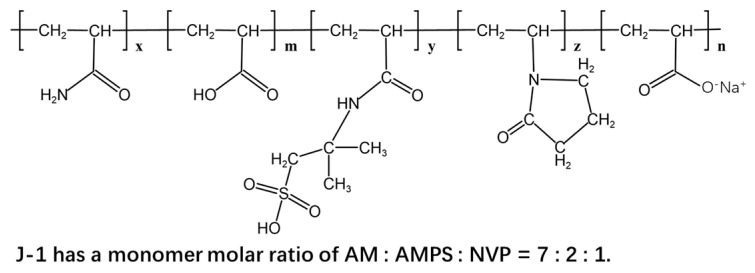
Structural formula of polymer J-1.

The precise chemical composition of polymer J-1 was determined using ^13^C NMR spectroscopy. As shown in Figure 4, key characteristic peaks were identified, including the resonance of the methylene group (–CH_2_) adjacent to the carbonyl in the NVP unit at δ = 77.3 and the methyl group (–CH_3_) in the AMPS unit at δ = 76.7. Through the comprehensive analysis and verification of the chemical shifts of various functional groups, the molecular structure of copolymer J-1 was finally determined as shown in Figure 5.

**Figure 6:**
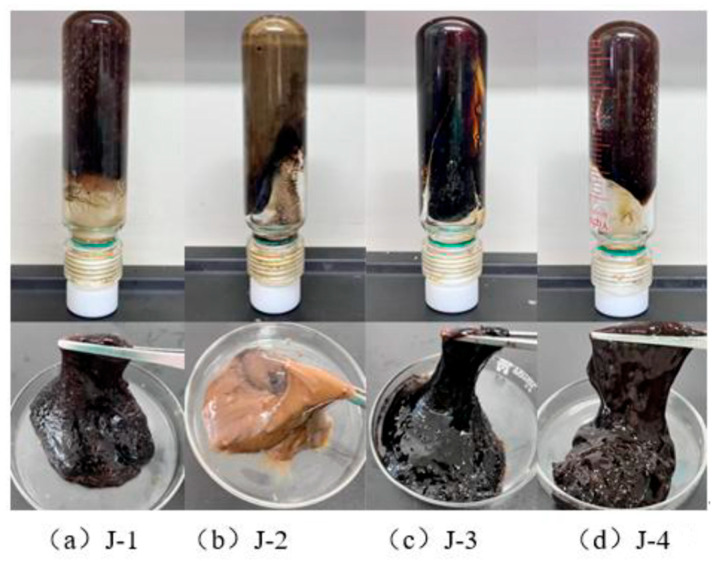
Different types of polymer crosslinking gel morphology.

**Figure 7:**
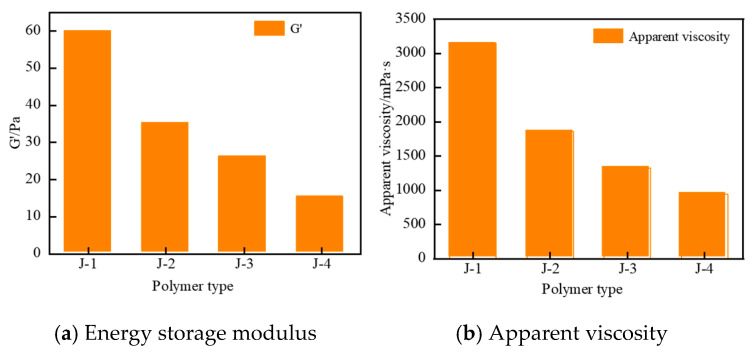
Comparison diagram of temperature resistance of different types of polymers.

Based on the above analysis, this section investigates the gelation performance of four polymers (J-1, J-2, J-3, and J-4). The gel systems were formulated with fixed concentrations of hexamethylenetetramine (0.6%), catechol (0.3%), and urea–formaldehyde resin (15%) and synthesized at 120 °C. As shown in Figure 6 and Figure 7, J-2 exhibits poor thermal stability, which prevents the formation of a high-strength gel. When J-3 is used as the main agent, the system shows a moderate increase in viscosity after 3 h of high-temperature aging, but the final gel strength remains low, reaching only a maximum grade of D. Although gel strength is partially improved with J-4 as the primary polymer, the resulting gel is markedly heterogeneous: approximately 60% of the system exhibits high strength, while the remaining 40% shows significantly lower structural integrity. Conventional HPAM-based systems are susceptible to structural breakdown under high-temperature conditions due to synergistic thermal degradation and hydrolysis of the polymer backbone, leading to ineffective crosslinking and insufficient gel strength. In contrast, when J-1 is employed as the main gel-forming agent, the resulting gel demonstrates high strength, significantly increased apparent viscosity, and excellent overall homogeneity. Therefore, J-1 is identified as the optimal polymer for the thixotropic gel plugging system.

**Figure 8:**
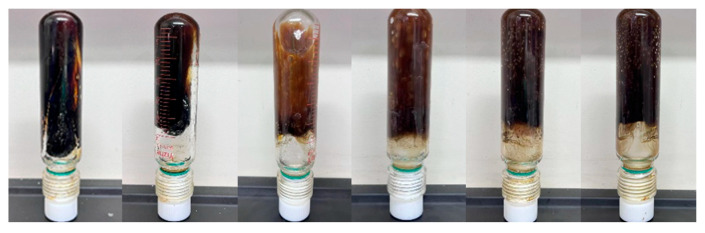
Gelatinization states of polymer J-1 gels at different concentrations.

**Figure 9:**
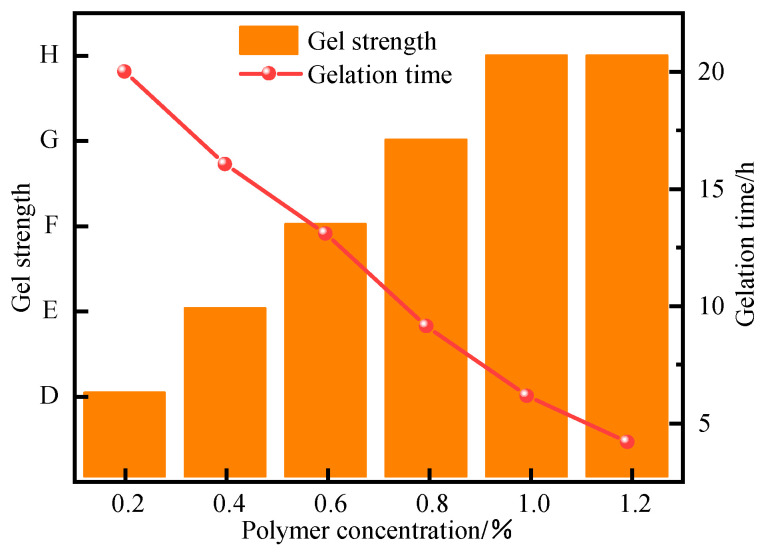
Effect of J-1 concentration on the strength of gel formation.

**Figure 10:**
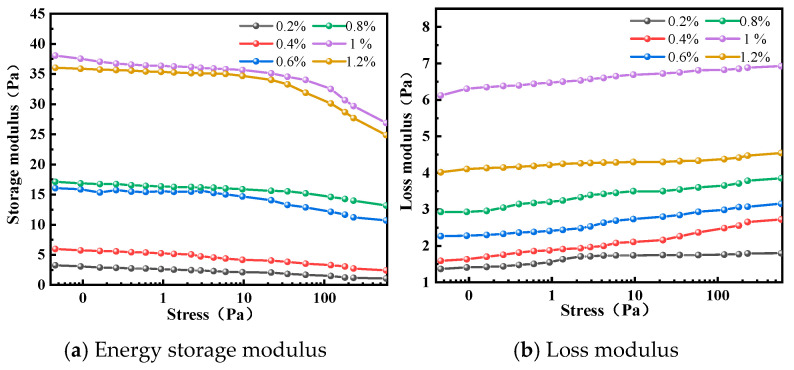
Effect of J-1 concentration on the complex modulus of a gel system.

The polymer concentration exerts a decisive influence on the gel system’s performance. An insufficient concentration fails to provide the necessary spatial density of molecular chains to establish effective crosslinking sites, resulting in a discontinuous network that either does not gel or forms a gel with excessively low strength. Conversely, an excessively high concentration causes a rapid increase in apparent viscosity and a non-linear rise in shear stress, adversely impacting the gel’s injectivity and flow properties. Furthermore, it accelerates the gelation rate, making it challenging to pump the system to the target wellbore location before it sets. To optimize these properties, the effect of polymer concentration (ranging from 0.2% to 1.2%) was investigated. As demonstrated in Figure 8, Figure 9 and Figure 10, the gel strength progressively increased from grade D at 0.2% to grade H at 1.0%. Concurrently, the gelation time decreased significantly from 20 h to 4 h over the same concentration range, indicating a strong dependence of the curing kinetics on polymer concentration.

Polymer concentration exerts a decisive influence on gel system performance. An insufficient concentration results in inadequate spatial distribution of molecular chains to form effective crosslinking sites, leading to a discontinuous network structure that either fails to gel or yields excessively weak gel strength. Conversely, excessively high concentrations cause a sharp rise in apparent viscosity, resulting in non-linear growth of shear stress that compromises injectability and accelerates gelation, making it difficult to pump the system to the target wellbore while still fluid. Experimental results (Figure 8, Figure 9 and Figure 10) demonstrate that as polymer concentration increases from 0.2% to 1.0%, gel strength progressively improves from grade D to grade H, while gelation time decreases from 20 h to 4 h. This enhancement is attributed to the increased availability of crosslinkable sites, which accelerates the crosslinking reaction. Beyond 1.0% concentration (e.g., at 1.2%), no further significant improvement in gel strength is observed, indicating complete reaction between the polymer and crosslinker at 1.0%. Rheological measurements confirm that at 1.0% concentration, the system achieves optimal viscoelastic properties with a storage modulus (G′) of 36 Pa and loss modulus (G″) of 6.5 Pa, exhibiting superior gel strength, structural integrity, and viscoelasticity. In conclusion, a polymer concentration of 1.0% is recommended as the optimal formulation for the J-1 type polymer in thixotropic polymer gel plugging systems.

**Figure 11:**
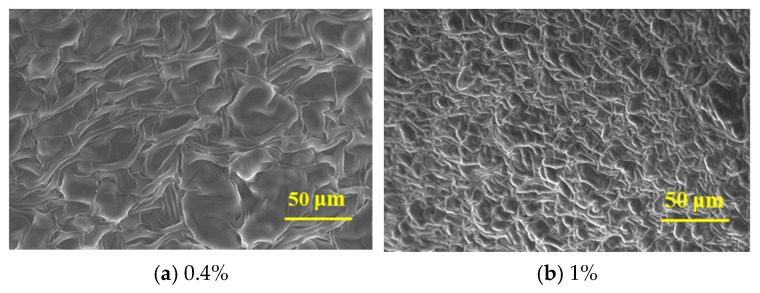
Microstructure of gel system at different J-1 concentrations.

Environmental scanning electron microscopy (ESEM) was employed to characterize the microstructure of the three-dimensional network formed by crosslinking polymer J-1 at varying concentrations (0.4%, 1%) with an organic phenol–formaldehyde system composed of hexamethylenetetramine and catechol. This analysis provides a mechanistic explanation for the gelation behavior of the system at the microscopic level. As shown in Figure 11, the density of the three-dimensional network markedly increases with higher polymer concentration, while the average mesh size within the network decreases. This microstructural evolution is attributed to the increased number of amide groups (–CONH_2_) present on the polymer chains at elevated concentrations. The higher density of amide groups raises the availability of active crosslinking sites, enhancing the reaction rate between the polymer and the phenol–formaldehyde crosslinker. As a result, a more compact and continuous network is formed, leading to a significant improvement in gel strength.

#### 2.2.2. Preferred Crosslinker Type and Concentration

In the formulation of gel systems, the crosslinking agent is the core functional component responsible for forming the three-dimensional gel network and significantly influences gelation performance. While organic phenol–formaldehyde crosslinking agents are currently widely used in polymer gel systems, conventional phenol-based formulations raise ecological toxicity concerns. Following the principles of green chemistry, this study compares the gelation time, gel strength, and long-term stability of polymer gels prepared with different crosslinking agents. It analyzes the effects of various types of phenolic and aldehyde crosslinkers on the gelation performance of polymer gel systems.

(1) Aldehyde Crosslinker Screening

This section analyzes the effects of different aldehyde crosslinking agents on the gelation performance of the gel system. The concentration of polymer J-1 was fixed at 1.0%, catechol at 0.3%, and urea–formaldehyde resin at 15%. Various aldehyde crosslinking agents were tested at concentrations ranging from 0.1% to 0.6%, and the gel system was prepared using deionized water. The gelation behavior was evaluated under high-temperature conditions (120 °C), focusing on the influence of aldehyde crosslinker type and concentration on gelation time and overall performance. The experimental results are summarized in Table 1.

To further evaluate the gelation behavior of the polymer gel system at 120 °C, the long-term stability influenced by different aldehyde crosslinkers was investigated. As summarized in Table 1, when formaldehyde was employed, gelation occurred within approximately 1 h due to its rapid release and high reactivity, leading to fast crosslinking and a quick increase in gel strength. However, this excessively fast reaction hindered the development of a tightly crosslinked network between the polymer and the crosslinker. Furthermore, after 6 h of high-temperature aging, severe dehydration and degradation of the polymer were observed. In the case of paraformaldehyde, structural limitations were evident during gelation. Although it hydrolyzes at high temperatures to release formaldehyde gradually, its release rate was insufficient to match the required crosslinking kinetics. Under these conditions, the polymer underwent chain scission and functional group degradation, reducing the availability of effective crosslinking sites. The slow-release behavior of paraformaldehyde at 120 °C impeded the formation of a stable crosslinked structure prior to polymer breakdown and hydrolysis. As a result, the system failed to form a coherent gel and completely hydrolyzed after 24 h of aging. Thus, neither formaldehyde nor paraformaldehyde is suitable for high-temperature gel plugging applications.

Finally, the gelation performance was assessed using HMTA as the aldehyde-releasing crosslinker. With the polymer J-1 concentration fixed at 1%, catechol at 0.3%, and urea–formaldehyde resin at 15%, the influence of HMTA concentration (0.2–0.8%) was evaluated at 120 °C. Within this range, the system formed robust gels with gelation times varying between 6 and 15 h.

Further investigation into the thermal stability revealed that at an HMTA concentration of 0.6%, the system demonstrated exceptional performance, exhibiting a dehydration rate below 10% after 7 days of aging at 120 °C. The gel also maintained stable viscoelastic properties under prolonged high-temperature exposure, with a storage modulus loss rate reduced by 62% compared to conventional crosslinking systems, indicating significantly enhanced network stability.

**Figure 12:**
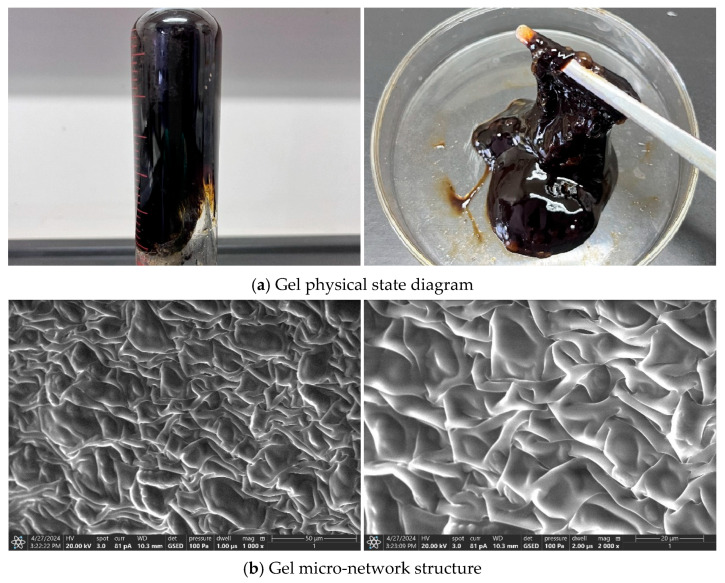
Schematic diagram of gel formation of polymer J-1/hexamethylenetetramine-catechol polymer gel system.

To elucidate the mechanism behind its improved thermal stability, environmental scanning electron microscopy (ESEM) was used to examine the gel’s microstructure post-gelation. As illustrated in Figure 12, the gel retained a continuous three-dimensional network structure even after extended high-temperature aging. This coherent architecture helps maintain structural integrity under high-temperature and high-pressure downhole conditions. Through this network-locking effect, the gel effectively mitigates excessive water loss, thereby delivering outstanding thermal stability.

**Figure 13:**
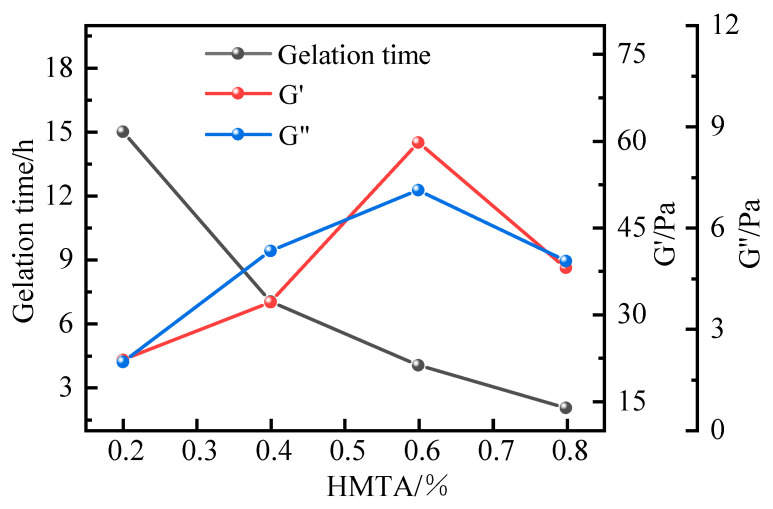
Effect of HMTA concentration on gel formation time and energy storage modulus.

In the study of high-temperature crosslinking kinetics, HMTA demonstrated a controlled thermal decomposition profile, leading to a moderate rate of formaldehyde release. This balanced release mechanism effectively synchronizes the crosslinking reaction with the thermal stability requirements of the polymer chains. Evaluation via the Sydansk bottle test method showed that the resulting gel possessed excellent integrity, achieving a strength grade of H. Based on these properties, HMTA was identified as the preferred aldehyde-releasing crosslinker. Using a fixed composition of 1% polymer J-1, 0.3% catechol, and 15% resin curing agent, the influence of HMTA concentration (0.2–0.8%) on system performance was further investigated. The experimental results are summarized in Figure 13.

**Figure 14:**
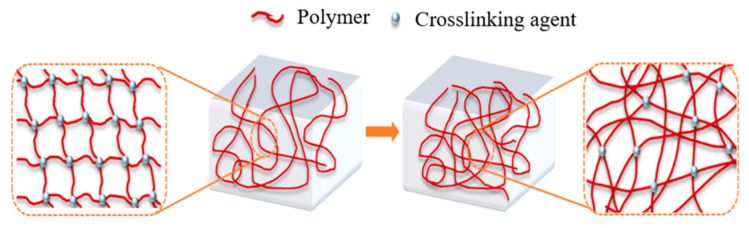
Schematic diagram of polymer gel over crosslinking.

Experimental results demonstrate that HMTA, within a concentration range of 0.2–0.8%, effectively crosslinks with the polymer at 120 °C, forming fully developed gel systems of varying strength. A clear inverse correlation was observed between HMTA concentration and gelation time, with overall gelation times spanning from 2 to 15 h. At an HMTA concentration of 0.2%, the gel system exhibited insufficient crosslinking, forming only a weak primary network (strength rating E), with storage and loss moduli of 22.3 Pa and 2.4 Pa, respectively. Increasing the HMTA concentration to 0.6% significantly raised the density of active crosslinking sites, resulting in substantially enhanced gel strength (strength rating H). At this concentration, the storage and loss moduli reached 52 Pa and 9 Pa, respectively, and the system displayed excellent viscoelastic properties. However, beyond 0.6%, a performance inflection point occurred, leading to reduced gel strength. This decline is attributed to excessive crosslinker concentration, which accelerated the reaction rate and promoted over-crosslinking. As a result, the proportion of network defects increased, hindering the formation of a stable crosslinked structure. This was evidenced by a 12–15% reduction in complex modulus, as shown in Figure 14. Based on performance optimization, the optimal HMTA concentration was determined to be 0.6%.

(2) Phenolic crosslinking agent preferred

This study systematically examines the effects of phenolic crosslinkers—phenol, hydroquinone, and catechol—on the gelation characteristics of polymer-based gels. Using ternary polymer J-1 (0.2–1.0%) as the primary gelling agent, with fixed concentrations of HMTA (0.6%) and urea–formaldehyde resin (15%), the research evaluates how type and dosage of phenolic crosslinkers modulate gel performance under high-temperature conditions. Experimental outcomes, including gel strength and crosslinking kinetics, are detailed in Table 2. The findings highlight distinct efficiency among the crosslinkers: catechol achieves optimal gel strength at lower concentrations, whereas phenol requires higher loadings for comparable performance. These insights provide practical guidance for tailoring crosslinked polymer gels in applications such as conformance control in high-temperature reservoirs.

As shown in Table 2, when phenol was used as the crosslinker, the resulting gel system formed an incomplete gel structure with insufficient strength, making it unsuitable for plugging applications. When hydroquinone was used as the phenolic crosslinker, a well-defined gel structure was formed, with a gelation time ranging from 8 to 15 h and a strength rating of D. However, its long-term stability was relatively poor. When the concentration of J-1 polymer increased to 0.8%, the gelation time of the system was reduced to 10 h. Further increasing the concentration to 1.0% shortened the gelation time to 7 h. Mechanistic analysis indicates that the increased molecular chain entanglement density facilitated the crosslinking reaction kinetics. Although the gelation time exhibited a decreasing trend, the gel strength significantly improved due to the enhanced efficiency of the three-dimensional network formation.

**Figure 15:**
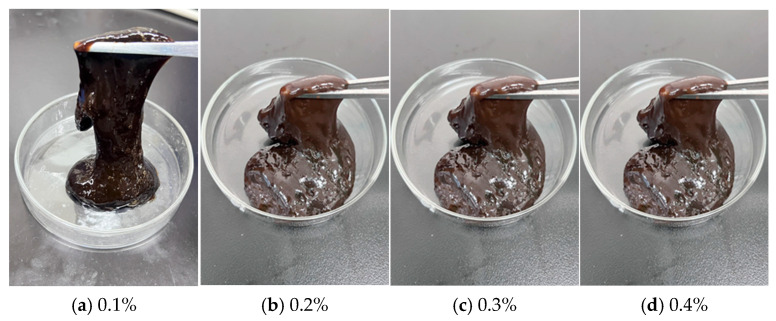
Gel-forming state of different catechol concentrations.

**Figure 16:**
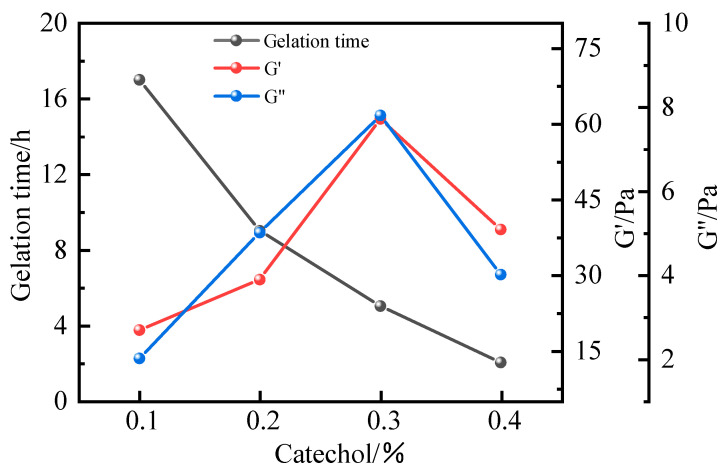
Effect of catechol concentration on gel formation time and energy storage modulus.

As shown in Figure 15 and Figure 16, when catechol was employed as the phenolic crosslinker, the resulting gel demonstrated superior strength compared to systems utilizing phenol or hydroquinone. The gel also exhibited enhanced wall adhesion and long-term stability, with a dehydration rate below 10% after 7 days of high-temperature aging. Based on these advantages, catechol was identified as the preferred crosslinker. Further investigation into the influence of catechol concentration revealed that gelation time decreased with increasing crosslinker content, while gel strength initially increased and subsequently decreased. At a concentration of 0.3%, the gel achieved optimal performance, with storage and loss moduli of 63 Pa and 9 Pa, respectively. Under these conditions, the gel displayed high viscosity and outstanding wall adhesion. However, when the catechol concentration exceeded 0.3%, both the composite modulus and gel strength declined. This behavior aligns with observations from earlier aldehyde crosslinker optimization experiments, where excessive crosslinker led to over-crosslinking and reduced gel quality. Thus, 0.3% catechol was established as the optimal concentration.

#### 2.2.3. Resin Curing Agent Type and Concentration Preference

To improve the long-term stability of the thixotropic polymer gel system under high-temperature reservoir conditions, this study incorporated urea–formaldehyde (UF) resin as a curing agent. When exposed to elevated temperatures, the UF resin undergoes self-polymerization and curing reactions. The reactive functional groups in UF resin molecules—such as –CH_2_OH (hydroxymethyl), –NH– (imino), and –NH_2_ (amino)—enable polycondensation reactions. Hydroxymethyl groups can form ether linkages (–CH_2_–O–CH_2_–) through dehydration condensation, while reactions between amino and hydroxymethyl groups yield methylene bridges (–CH_2_–NH–) with the release of water. As a result, UF resin molecules interconnect and assemble into an extensive three-dimensional network. This structure significantly enhances the gel’s strength, viscoelasticity, and thermal resistance, thereby improving its long-term stability. Such reinforced properties are especially advantageous for plugging large fracture leakage channels under high-temperature and high-pressure reservoir conditions.

Under high-temperature reservoir conditions, the gel plugging system may experience polymer chain breakage, changes in molecular aggregation states, and other phenomena due to temperature effects, which can impact the gelation performance of the gel system. The three-dimensional network framework formed by UF resin can effectively restrict the disordered motion of molecules within the gel system. At the same time, this three-dimensional network structure can enhance the gel’s pressure-bearing capacity, enabling it to withstand the formation pressure under high-temperature conditions. However, when the amount of UF resin is insufficient, an incomplete three-dimensional network structure may form, reducing the number of crosslinking points between molecules, which leads to insufficient strength in the gel system. In this case, the gel body formed by the gel plugging agent may deform or even rupture, making it unable to withstand the excessive pressure or formation fluid scouring forces in deep reservoirs, which affects the plugging effect.

Increasing the dosage of UF resin introduces more reactive functional groups, promoting the formation of a denser three-dimensional network. This leads to enhanced gel strength, improving its ability to withstand external pressure and fluid erosion, thereby enhancing the sealing efficiency in formation pores and fractures. However, excessive UF resin can cause the gel to become overly rigid and brittle. The high degree of crosslinking reduces elasticity, making the gel prone to cracking under mechanical stress and ultimately diminishing its plugging performance. Furthermore, higher resin content accelerates the curing rate, as more functional groups are available to participate in crosslinking reactions over a shorter period. This can result in an overly intense curing process that becomes difficult to control.

**Figure 17:**
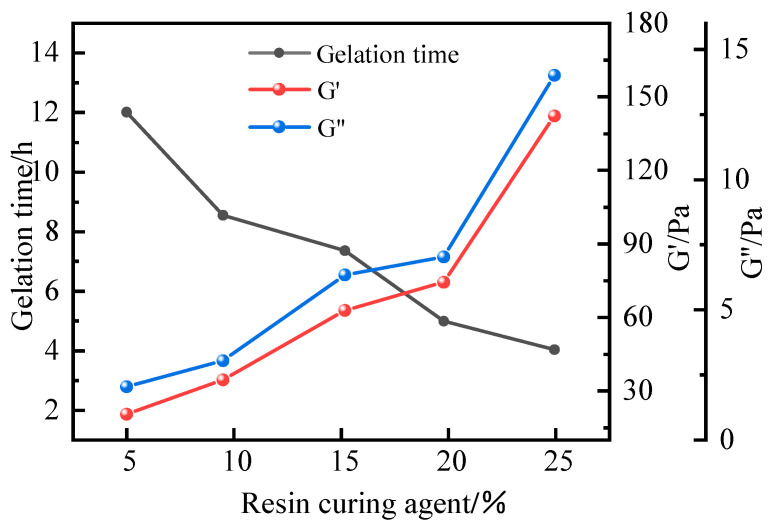
Effect of urea–formaldehyde resin concentration on gel formation time and energy storage modulus.

Based on the formulation with 1.0% polymer J-1, 0.6% HMTA, 0.3% catechol, and gelation at 120 °C, the effect of urea–formaldehyde (UF) resin concentration on gel strength was investigated. As shown in Figure 17, UF resin forms a stable gel within the concentration range of 5% to 25% under high-temperature conditions. Increasing the UF resin content significantly enhances the gel strength and shortens the gelation time, which ranges from 4 to 12 h. At 15% UF resin, the gel demonstrates optimal performance with a storage modulus of 62.4 Pa and a loss modulus of 6.3 Pa, meeting the requirements for effective plugging. However, exceeding this concentration leads to excessive crosslinking, resulting in reduced elasticity, pronounced dehydration, and poor controllability of the curing process. Therefore, a UF resin concentration of 15% is considered ideal for achieving a balance between gel strength and manageable gelation behavior.

### 2.3. Structural Characterization of Polymer Gel Systems

**Figure 18:**
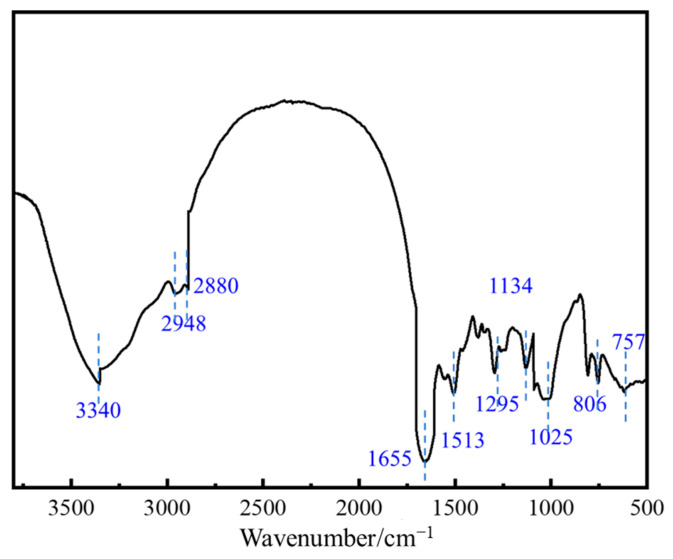
Infrared structure characterization of polymer gel system.

This study employs Fourier transform infrared spectroscopy (FTIR) to characterize the molecular structure of the thixotropic polymer gel system. The vibrational information in the spectral range (1310~20 cm^−1^) and functional group region (3500~350 cm^−1^) reveals the characteristic chemical bonding within the system. The test results shown in Figure 18 indicate that the broad and strong absorption peak at 3340 cm^−1^ corresponds to the stretching vibration mode of the amino group (–NH_2_) or amide group (–COHN_2_); the characteristic peak at 2948 cm^−1^ can be attributed to the antisymmetric vibration of carboxylate (–COO^−^) or sulfonate (–SO_3_^−^) groups. The characteristic vibration signal of the aliphatic C–H bond is observed at 2880 cm^−1^, while the twin peaks at 1655 cm^−1^ and 1513 cm^−1^ jointly indicate the stretching vibration of the C=O group in the amide moiety. A strong peak at 1295 cm^−1^ corresponds to the deformation vibration of methylene/methyl groups (–CH_2_–/–CH_3_), and a prominent absorption at 1134 cm^−1^ confirms the presence of the C–O functional group. The peak at 1025 cm^−1^ is associated with S=O bond vibration. In the low-frequency region, the doublet at 806 cm^−1^ and 757 cm^−1^ displays a distinctive “split peak” profile, characteristic of the in-plane bending vibration of the S–O–H bond in sulfonic acid groups (–SO_3_H).

The Fourier transform infrared spectroscopy (FTIR) characterization results confirm that the phenolic crosslinker and polymer J-1 interact through a synergistic mechanism of hydrogen bonding and covalent crosslinking, forming a three-dimensional network with multiscale interactions. This imparts the gel system with unique thixotropic responsiveness and thermal stability. Analysis of the characteristic absorption peaks reveals changes in the vibrational modes of the phenolic hydroxyl group (3210–3590 cm^−1^) and the amide group (around 1650 cm^−1^), suggesting the formation of intermolecular hydrogen bonding and covalent crosslinking reactions. This confirms the successful synthesis of the target thixotropic polymer gel system. These structural insights provide a molecular-level mechanistic basis for subsequent rheological investigations and practical engineering applications.

Nuclear magnetic resonance (NMR) spectroscopy plays a critical role in characterizing the crosslinked network structure of thixotropic polymer gels. The technique relies on the spin properties of atomic nuclei: when placed in a magnetic field, nuclei in distinct chemical environments absorb radiofrequency radiation at characteristic frequencies. This absorption causes excitation and transitions within the nuclear spin system, resulting in detectable induced electromotive force signals. Information such as chemical shifts, peak splitting, and integration areas of these signals provides detailed insights into the molecular structure of the gel system. For crosslinked polymer gels, NMR can provide information about atoms near the crosslinking points. For example, by analyzing the signals from specific atoms on the crosslinker, one can understand the crosslinking density and structure.

**Figure 19:**
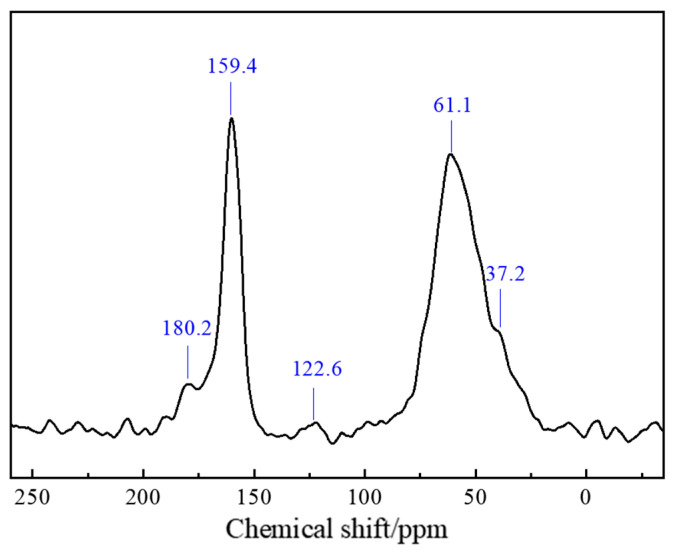
Nuclear magnetic resonance analysis of polymer gel system.

Carbon-13 nuclear magnetic resonance (^13^C-NMR) relies on the magnetic resonance properties of ^13^C nuclei. When subjected to a magnetic field, these nuclei occupy discrete energy levels. Radiofrequency pulse excitation induces transitions between these energy levels, resulting in measurable nuclear magnetic resonance signals. The resonance frequency is influenced by the chemical environment of each ^13^C nucleus. These frequencies are converted into chemical shifts (expressed in ppm), which provide detailed structural insights. The chemical shift (δ) serves as a fundamental parameter that reflects electron density distribution, thereby revealing structural characteristics of individual carbon atoms within the polymer gel network. Nuclear magnetic resonance analysis of the polymer gel was performed to determine the specific chemical structure of the gel. The ^13^C-NMR spectrum of this thixotropic polymer gel system is shown in Figure 19. In the figure, the peak at δ = 180.2 corresponds to the C=O bond in the amide group, the peak at δ = 159.4 corresponds to the carbon atoms in the benzene ring structure, the peak at δ = 61.1 corresponds to the –NH_2_ or –COHN_2_ groups in the polymer structure, and the peak at δ = 37.2 corresponds to the chemical shift of the –CH_3_ group in the ATBS structure.

### 2.4. The Working Performance of the Gel–Resin Coagulation Composite System

**Figure 20:**
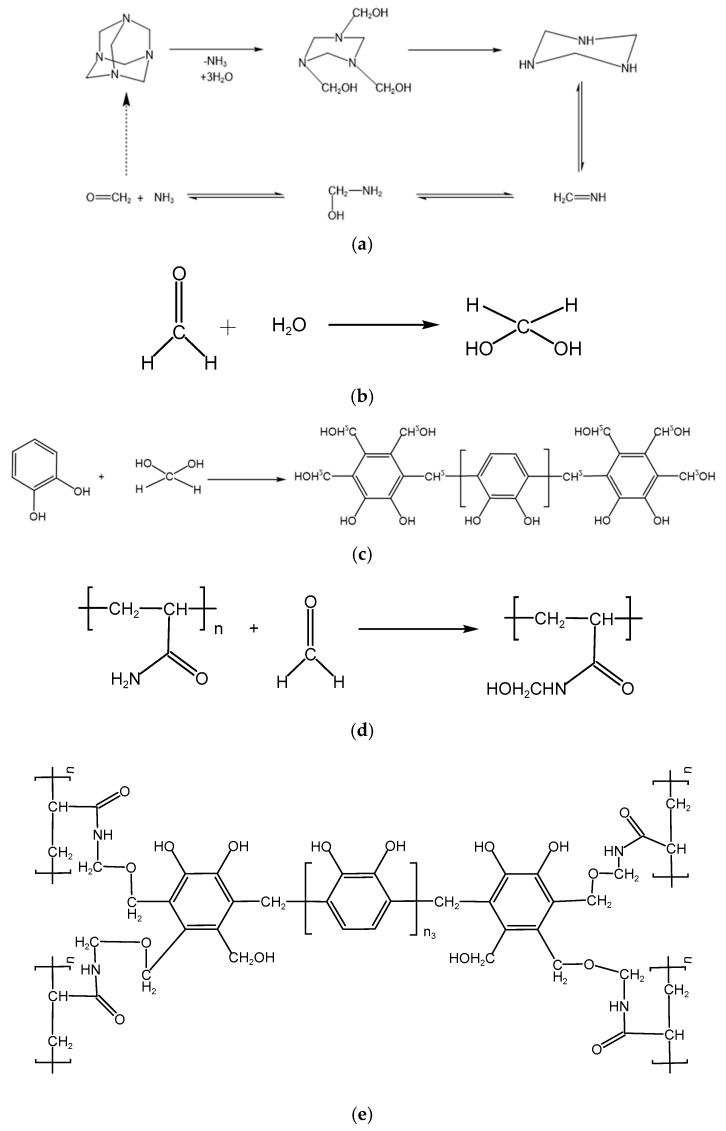
Crosslinking reaction mechanism analysis diagram. (**a**) Hexamethylene tetramine (HMTA) gradually decomposes to form formaldehyde and ammonia, (**b**) Formaldehyde forms the active intermediate methylene glycol through hydration, (**c**) Etherification-Condensation Cascade Reaction of Methylene Diol and Catechol, (**d**) Hydroxymethylation modification, (**e**) Synthesis of gel by dehydration condensation reaction.

Based on the regulation patterns of polymer gel formation performance by different phenol–formaldehyde crosslinking agent systems (as detailed in Section 2.2.2), this subsection provides an in-depth analysis of the gelation mechanism. Taking the hexamethylenetetramine (HMTA) crosslinking system as an example, under high-temperature (>100 °C) and acidic conditions (pH = 4~6, controlled by 5% dilute hydrochloric acid), HMTA undergoes gradual thermal decomposition to generate formaldehyde and ammonia gas, as shown in the reaction equation in Figure 20a. The released formaldehyde is then hydrated to form the active intermediate methylene glycol, as shown in the reaction equation in Figure 20b. Next, methylene glycol molecules undergo an etherification–condensation cascade reaction with catechol, sequentially forming the catechol-1,2-dimethyl ether intermediate and some phenol–formaldehyde resin crosslinking agents, as shown in the reaction equation in Figure 20c. Meanwhile, some –CONH_2_ groups on polyacrylamide (HPAM) react with the more reactive methylene glycol to undergo hydroxymethylation modification, as shown in the reaction equation in Figure 20d. Finally, the phenol–formaldehyde system and hydroxymethylated HPAM synthesize the gel system via a dehydration condensation reaction, as shown in the reaction equation in Figure 20e.

### 2.5. Thixotropic Properties of Polymer Gel Systems

In rheology, thixotropy describes a material’s time-dependent shear thinning behavior. When subjected to shear stress, the breakdown of the material’s internal structure occurs more rapidly than its recovery, resulting in hysteresis in the viscosity curve. During gel formation, this structural network develops through crosslinking interactions between polymer chains and crosslinking agents. The evolution of this microstructure can be quantitatively characterized using thixotropic loop measurements under oscillatory shear conditions.

To determine whether a polymer solution is thixotropic, a stepped ramp test (up-ramp and down-ramp) must be performed. In this test, the shear rate is gradually increased from zero to a maximum value and then immediately decreased back to zero at the same rate, producing a flow curve that relates shear stress to shear rate. When the upward ramp and downward ramp curves do not coincide and instead form a closed loop, this indicates the presence of a hysteresis loop—a characteristic signature of thixotropic behavior. Fluids with thixotropic properties exhibit this hysteresis loop phenomenon during the acceleration and deceleration processes due to differences in the disruption and reconstruction of the internal structure. During the acceleration process, as the shear rate increases, the internal structure of the fluid is gradually disrupted, leading to a corresponding increase in shear stress. However, as the structure continues to be disrupted and resistance decreases, the rate of increase gradually diminishes. During the deceleration process, the fluid structure begins to rebuild, and shear stress decreases. However, since the reconstruction of the structure takes time, the deceleration curve lies below the acceleration curve, forming the hysteresis loop.

Due to their sensitivity to handling history, thixotropic solutions require careful preconditioning. To ensure reproducible results, the sample was subjected to pre-shearing at 10 s^−1^ prior to measurement. The test protocol involved a shear rate ramp from 10 s^−1^ to 200 s^−1^ (upward curve) followed by an immediate ramp back to 0 s^−1^ (downward curve). The resulting hysteresis loop demonstrates the structural hysteresis between deformation and recovery under cyclic shear. The selected gel system exhibited a significantly larger hysteresis area compared to the base polymer solution, confirming its superior thixotropic performance. During the acceleration process, as the shear rate rapidly increases, the fluid viscosity decreases quickly, indicating that the structure of the solution is rapidly disrupted under shear. During the deceleration process, even though the shear rate decreases, the viscosity of the fluid does not immediately return to its original state due to the time required for structural reconstruction, which also reflects the time-dependent behavior of thixotropic fluids.

**Figure 21:**
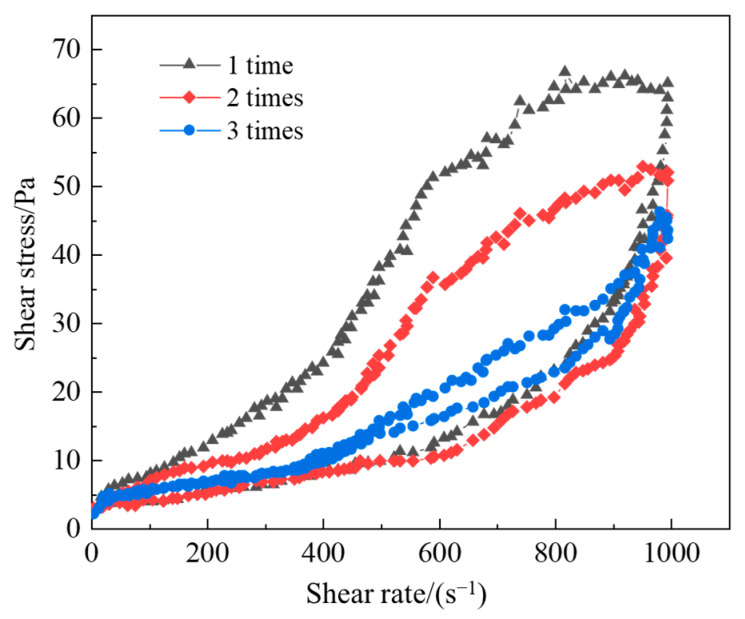
Different hysteresis zones of gel solutions during cyclic testing.

Different shear rate ranges result in varying thixotropy loop areas. If the test is conducted within a lower shear rate range, the internal structure of the fluid may only be slightly disrupted, resulting in a smaller thixotropy loop area. In higher shear rate ranges, the degree of structural disruption is greater, leading to an increased thixotropy loop area. Shear history is also crucial. If the fluid has undergone multiple shear cycles in advance, its internal structure may have undergone a certain degree of alteration. A cyclic shear experiment was performed on the selected gel solution, and the results are shown in Figure 21. The experimental results show that the area of the first thixotropic ring is about 6574 Pa·s^−1^, the area of the second thixotropic ring is about 4276 Pa·s^−1^, and the area of the third thixotropic ring is about 1623 Pa·s^−1^. In several shear cycles, the hysteresis loop of the first cycle is clearly larger than that of the subsequent cycles, while in the later cycles, the characteristics of the thixotropy loop resemble the first one. After three cycles, the hysteresis area gradually decreases, and the ascending and descending curves almost overlap. Furthermore, after each cycle, the hysteresis continues to diminish. This occurs because, for polymer solutions, long-chain polymer molecules tend to become entangled in their initial state. During shear, as the shear rate increases, these entanglements are gradually unraveled. During the cyclic shear process, if the molecular chains are excessively stretched or irreversibly broken by bond cleavage, the chains will struggle to return to their original entangled state, resulting in a reduction in the fluid’s thixotropic properties and a smaller thixotropy loop area.

### 2.6. High-Temperature Gel Formation Performance of Polymer Gel System

**Figure 22:**
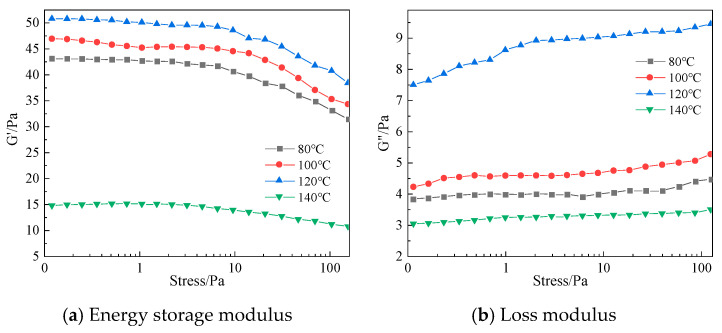
Effect of different temperatures on the composite modulus of gel systems.

The gelation time of the polymer gel system is highly sensitive to temperature. In general, higher temperatures accelerate chemical reaction kinetics. For chemically crosslinked polymer gel systems—such as those formed via condensation reactions between polymers and crosslinking agents—elevated temperatures promote faster reaction rates between the components. This increase in temperature raises the frequency of effective molecular collisions, thereby accelerating the crosslinking process and shortening the gelation time. High temperature also considerably influences the gel strength. Increased thermal energy can promote greater extension of polymer chains, strengthening intermolecular interactions. At suitably high temperatures, this enhanced mobility allows polymer chains to entangle and crosslink more effectively, leading to improved gel strength. However, excessively high temperatures may disrupt the three-dimensional network of the gel. Beyond a certain threshold, intensified molecular motion can break chemical bonds and degrade crosslinking sites, compromising structural integrity. To examine the impact of temperature on gelation time and gel strength, experiments were conducted using an optimized formula designed for high-temperature subsurface applications. The gel system was evaluated across a temperature range of 80 °C to 140 °C. The corresponding results are presented in Figure 22.

According to the data in Figure 22, the storage modulus varies significantly at different temperatures and decreases with increasing stress. The higher the temperature, the lower the storage modulus. The loss modulus increases with increasing stress, with relatively smaller differences across temperatures. Moreover, under consistent stress conditions, the storage modulus (G′) of the gel system consistently remains significantly higher than its loss modulus (G″). This indicates dominant elastic behavior over viscous flow, confirming the formation of a robust, solid-like network. Such strong elastic properties are advantageous for achieving effective plugging in lost circulation zones, as the gel can withstand deformation and maintain structural integrity under downhole stresses.

When the gelation temperature is set at 120 °C, the system reaches the optimal crosslinking state and the complex modulus reaches its maximum value. Dynamic frequency scan data show that the storage modulus and loss modulus stabilize at 50 Pa and 9 Pa, respectively, indicating the formation of a high-strength three-dimensional network gel body at this temperature, with optimal viscoelastic response. When the temperature is raised to 140 °C, the accelerated molecular thermal motion and increased collision reactions between the polymer macromolecules and phenolic resin crosslinking agents lead to a reduction in the crosslinking structure density, thereby decreasing the gel’s strength.

## 3. Materials and Methods

### 3.1. Experimental Material

#### 3.1.1. Experimental Chemicals and Materials

The chemicals and materials used in the experiment are shown in Table 3. The J-1, J-2, J-3, and J-4 mentioned in the table are random copolymers based on acrylamide with different molecular weights and compositions, all supplied by Shandong Noor Chemical Co. (Jining, China).

The polymer J-1 was synthesized by dissolving AM, AMPS, and NVP in a molar ratio of 7:2:1, followed by adding 0.3% free radical initiator benzoyl peroxide (BPO) under stirring. The reaction mixture was placed in a water bath at 60–80 °C for 6 h until the solution became highly viscous. The polymer was then precipitated in ethanol, filtered, and dried to obtain the final product J-1. The polymer J-2 was synthesized by dissolving AM and AMPS in a molar ratio of 8:2 in an appropriate amount of solvent. After complete dissolution, 0.1% potassium persulfate (KPS) was added as a free radical initiator under continuous stirring. The reaction was carried out in a water bath maintained at 60–80 °C for 4 h until the solution became highly viscous. The resulting polymer was then precipitated in ethanol, followed by filtration and drying to obtain the final product J-2.

The hydrolyzed polyacrylamides J-3 and J-4 were synthesized by first completely dissolving an appropriate amount of AM monomer in deionized water, followed by the addition of 0.5% ammonium persulfate (APS) as a free radical initiator under stirring. The solution was reacted at 60 °C under nitrogen protection for 5 h to form high-molecular-weight polyacrylamide (PAM) gel. A defined amount of sodium hydroxide (NaOH) solution was then slowly added to the gel under stirring, and controlled hydrolysis was conducted at 90 °C for 3 and 4 h, respectively, to achieve a degree of hydrolysis (DH) between 20 and 25%. After the reaction, the mixture was neutralized to pH 7 with hydrochloric acid, precipitated in excess acetone, and then filtered, washed, and dried to obtain the low-molecular-weight products J-3 and J-4.

#### 3.1.2. Experimental Instruments

The main apparatus used in the experiment is shown in Table 4.

### 3.2. Experimental Methods

#### 3.2.1. Preparation of Polymer Solutions

The preparation and dilution steps of the polymer stock solution are as follows: First, the vacuum-dried polymer powder (J-1, J-2, J-3, J-4 are all provided by Shandong Noor Chemical Co.) was accurately weighed, and deionized water was used as the solvent. The polymer powder was added incrementally to prepare a 1.0% stock solution. During the preparation, a magnetic stirrer (set to 300 ± 50 rpm, 25 ± 0.5 °C) was used for continuous stirring for no less than 6 h to ensure complete dissolution, followed by standing at room temperature (25 °C) for 24 h. Gradient dilution is performed using deionized water from the same batch, with each dilution stage thoroughly mixed using the stirrer, ultimately obtaining polymer solutions with a series of concentrations for testing.

#### 3.2.2. Evaluation Methods for Gelation Time and Gelation Efficacy of Polymer Gel Systems

**Figure 23:**
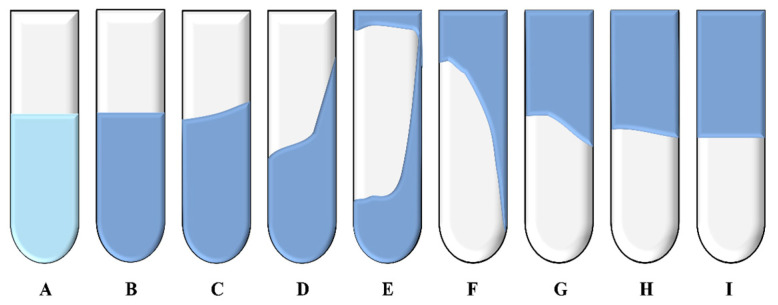
Schematics of different gel strength codes.

The prepared polymer gel precursor solution was slowly poured into a temperature- and pressure-resistant bottle and placed in a thermostatic air-drying oven, allowing the polymer and phenolic resin crosslinker to undergo crosslinking reactions under high-temperature conditions. The Sydansk bottle test method was used to determine the gelation time and gelation strength [25]. The time at which the gel state remains unchanged is recorded as the gelation time (GT). Based on the gel’s inverted hanging state and tongue-out state, the gel system is divided into 9 levels: A (discontinuous state), B (highly fluid state), C (fluid state), D (moderately fluid state), E (difficult to flow state), F (highly deformed, non-flowing state), G (moderately deformed, non-flowing state), H (slightly deformed, non-flowing state), I (rigid state). A schematic diagram of the different strength levels is shown in Figure 23.

#### 3.2.3. Polymer Gel System Strength Test Method

A plate rheological testing platform based on the HAKKE Mars60 rheometer (Thermo Fisher Scientific Inc., Waltham, MA, USA) was used to characterize the rheological properties of the polymer gel system. The experimental setup selected a P35 titanium alloy coaxial rotor, with the plate gap set to 52 μm using the control module. Dynamic rheological analysis mode was used, with steady-state shear scanning and dynamic oscillation frequency scanning conducted in dual-mode tests. The frequency was set at 1 Hz, while the shear rate was increased from 0.1 s^−1^ to 100 s^−1^ and the shear stress from 0.1 Pa to 100 Pa. This protocol was designed to evaluate the structural integrity and viscoelastic behavior of the gel network under deformation.

#### 3.2.4. Microstructure Analysis of Polymer Gel System

In this study, a JSM-7200 F field emission environmental scanning electron microscope (FE-SEM) (JEOL Ltd., Akishima, Japan) was used to characterize the microstructure of the gel network. The gel samples were first treated with liquid nitrogen to fix the three-dimensional network structure, followed by vacuum freeze-drying to sublimate the water directly from the sample into gas. Conductive adhesive tape was used to attach the sample to the stage, and gold powder was sprayed onto the surface to improve conductivity. The sample was placed in the chamber of an environmental scanning electron microscope, and images at various magnification levels were captured at room temperature using specialized software. An image featuring a white scale bar representing 10 μm at the bottom center was selected for analysis.

#### 3.2.5. Infrared Spectral Characterization of Polymer Gel Systems

The molecular structures of the initial polymers (J-1 to J-4) and the gel system were analyzed using an FTIR-7600 Fourier Transform Infrared Spectrometer (Shanghai Precision Instrument Co., Ltd.). For the initial polymers, 1.0 mg of vacuum-dried polymer powder was homogenized with 99.0 mg of dry KBr and pressed into a transparent pellet under 10 MPa. For the gel system, 1.0 mg of freeze-dried gel (lyophilized to eliminate moisture interference) was similarly prepared as a KBr pellet using the above method. FTIR spectra were acquired over the range of 400–4000 cm^−1^ at a resolution of 4 cm^−1^ with 35 accumulated scans. Characteristic absorption peaks—such as N–H stretching at 3419 cm^−1^ and S=O symmetric stretching at 1107 cm^−1^—were used to identify functional groups.

#### 3.2.6. Thermogravimetric Analysis of Polymer Gel Systems

The thermal stability of the gel molecular structure was evaluated using a thermogravimetric analyzer (TGA 2 SF, Mettler Toledo, Zurich, Switzerland). The instrument was first switched on and preheated, and relevant experimental parameters—including temperature and heating rate—were configured. An empty crucible was then placed inside, and the mass was tared. Finally, a suitable amount of sealant was introduced into the instrument. For all samples, the measurement pressure was set to 300 Pa, the gas flow rate was maintained at 20 cm^3^/min, the heating rate was 10 °C/min, and the temperature was scanned from 30 to 600 °C.

#### 3.2.7. NMR Analysis of Polymer Gels

Nuclear magnetic resonance (NMR) spectroscopy was used to characterize the molecular structures of the polymer and its gel system. The sample was dissolved in a suitable deuterated solvent and transferred into a standard NMR tube. Prior to measurement, the magnetic field homogeneity was optimized by performing standard shimming procedures. A pulse sequence was selected and configured according to the desired NMR experiment. Carbon-13 (^13^C NMR) spectra were then acquired under appropriate acquisition parameters to enable structural analysis.

## 4. Conclusions

(1) Conventional acrylamide-based monomers exhibit low thermal stability. In this study, the polymer J-1, prepared by the ternary copolymerization of AM, AMPS, and NVP, is investigated. The incorporation of AMPS into the polymer molecular structure, with its large side groups on the main chain, significantly enhances the polymer’s thermal stability. The polymer J-1 incorporates both AMPS and NVP as building blocks into the polymer chain, further improving its thermal stability.
(2) HMTA can stably release active formaldehyde molecules under high-temperature conditions, which not only avoids the issue of excessively fast crosslinking rates caused by the direct use of formaldehyde but also overcomes the problem of the low release rate of paraformaldehyde. This controllable release property allows the crosslinking reaction and the thermal degradation process of the polymer chains to reach a dynamic balance, effectively optimizing the crosslinking rate of the gel system.
(3) The ortho-hydroxyphenol structure of catechol can form multi-functional crosslinking sites, reacting with formaldehyde to generate high-density crosslinked clusters. Compared to the phenol system, this significantly enhances the three-dimensional network density of the gel, thereby improving the gel strength and structural stability of the system.
(4) After introducing the resin curing agent urea–formaldehyde resin into the gel system, the in situ generated resin network structure provides a rigid framework for the gel system. This dual-network synergistic effect not only enhances the gel’s thermal resistance and gel strength but also improves the gel sealant’s adaptability to block large crack leakage channels, maintaining long-term stable plugging performance.
(5) A single-factor experimental method was used to investigate the effects of polymer type, crosslinker type, and concentration on the gel system. Based on the experimental results, the optimal system composition was determined to be 1% polymer J-1 + 0.3% catechol + 0.6% HMTA + 15% urea–formaldehyde resin.
(6) A cyclic shear experiment was conducted to evaluate whether the polymer solution exhibits thixotropic behavior. Under the cyclic shear rate, a hysteresis between the solution’s degradation and reconstruction was observed. The selected thixotropic gel system had a larger hysteresis area compared to the polymer mother liquid, indicating excellent thixotropic performance before gel formation when the system is in a gel-like state.
(7) The selected thixotropic polymer gel system can form a complete gel system within the temperature range of 80 °C to 140 °C, and the gelation time decreases as the temperature increases. The gel’s viscosity at 120 °C is 7500 mPa·s, with a storage modulus and loss modulus of 51 Pa and 6 Pa, respectively.

## Figures and Tables

**Table 1:** Effect of different aldehyde crosslinkers on gel effect.

Aldehyde Crosslinking Agent and Concentration (%)		Catechol Concentration (%)	Gumming Time (h)	Glue Forming Effect and Stability
formaldehyde	0.1	0.3	2	After the forming strength reaches G, it is degraded at high temperature for 6 h
	0.3		1	After the forming strength reaches G, it is degraded at high temperature for 6 h
	0.6		1	After the forming strength reaches G, it is degraded at high temperature for 5 h
triformaldehyde	0.1	0.3	fail to gel	—
	0.3		fail to gel	—
	0.6		fail to gel	—
HMTA	0.1	0.3	15	Gel strength G, 7 days dehydration less than 10%
	0.3		11	Gel strength H, 7 days dehydration less than 10%
	0.6		6	Gel strength H, 7 days dehydration less than 10%

**Table 2:** Effect of different phenolic crosslinkers on gel formation effect.

Crosslinking Agent and Concentration (%)	Polymer Concentration (%)	Gelation Time (h)	Gelling Effect and Stability
0.3% phenol + 0.6%HMTA	0.2	—	Strength is too weak
	0.4	—	
	0.6	—	
	0.8	—	
	1.0	—	
0.3% Hydroquinone + 0.6%HMTA	0.2	15	The gumming strength D, 7 days dehydration less than 35%
	0.4	14	The gumming strength D, 7 days dehydration less than 35%
	0.6	12	The gumming strength D, 7 days dehydration less than 35%
	0.8	10	The gumming strength E, 7 days dehydration less than 30%
	1.0	8	The gumming strength E, 7 days dehydration less than 30%
0.3% Catechol + 0.6%HMTA	0.2	16	The gumming strength D, 7 days dehydration less than 10%
	0.4	14	The gumming strength D, 7 days dehydration less than 10%
	0.6	11	The gumming strength E, 7 days dehydration less than 10%
	0.7	8	The gumming strength F, 7 days dehydration less than 10%
	0.8	6	The gumming strength H, 7 days dehydration less than 10%

**Table 3:** Experimental drugs and materials.

Number	Name	Concentration/%	Manufacturer
1	J-1	—	Shandong Noor Chemical Co. (Jining, China)
2	J-2	—	Shandong Noor Chemical Co. (Jining, China)
3	J-3	—	Shandong Noor Chemical Co. (Jining, China)
4	J-4	—	Shandong Noor Chemical Co. (Jining, China)
5	formaldehyde	AR	Shanghai Aladdin Reagent Co. (Shanghai, China)
6	trimethylformaldehyde	AR	Shanghai Aladdin Reagent Co. (Shanghai, China)
7	HMTA	AR	Shanghai McLean Biochemical Technology Co. (Shanghai, China)
8	catechol	AR	Shanghai McLean Biochemical Technology Co. (Shanghai, China)
9	hydroquinone	AR	Shanghai McLean Biochemical Technology Co. (Shanghai, China)
10	catechol	AR	Shanghai McLean Biochemical Technology Co. (Shanghai, China)
11	UF resin	—	Zibo Ocean Industry Co. (Zibo, China)
12	deionized water	—	—
13	dilute hydrochloric acid	5%	—

**Table 4:** Main instruments.

Number	Instrument Name	Manufacturer
1	TGA/DTAThermogravimetric Analyzer	Mettler Toledo Technology Co., Ltd. (Shanghai, China)
2	High-temperature and high-pressure plugging and displacement device	Nantong Xinhua Cheng Scientific Research Instruments Co., Ltd. (Nantong, China)
3	Quanta 200F Field Emission Scanning Electron Microscope	FEI (Hillsboro, OR, USA)
4	HAKKER Mars60 Rheometer	Thermo Fisher (Dreieich, Germany)
5	Fourier Transform Infrared Spectrometer FTIR-7600	Shanghai Precision Instrumentation Co., Ltd. (Shanghai, China)
6	Constant temperature blast drying oven	Shanghai Senxin Experimental Instrument Co., Ltd. (Shanghai, China)
7	High-temperature and high-pressure water loss meter	Jinan New Test King Testing Machine Co., Ltd. (Jinan, China)
8	Visual crack modeling	Nantong Xinhua Cheng Scientific Research Instruments Co., Ltd. (Nantong, China)
9	magnetic stirrer	Shanghai Ni Yue Instrument Co., Ltd. (Shanghai, China)

## Data Availability

Data are contained within the article.
